# Detection of Imatinib Based on Electrochemical Sensor Constructed Using Biosynthesized Graphene-Silver Nanocomposite

**DOI:** 10.3389/fchem.2021.670074

**Published:** 2021-04-22

**Authors:** Zhen Wu, Jingjing Liu, Minmin Liang, Haoyue Zheng, Chuansheng Zhu, Yan Wang

**Affiliations:** ^1^Day Chemotherapy Unit, Qianfoshan Hospital, The First Affiliated Hospital of Shandong First Medical University, Jinan, China; ^2^Hematology Department, Qianfoshan Hospital, The First Affiliated Hospital of Shandong First Medical University, Jinan, China; ^3^Shandong First Medical University, Jinan, China

**Keywords:** biosynthesis, imatinib, silver nanoparticle, graphene composite, *Lycoris longituba*

## Abstract

The establishment of a monitoring technique for imatinib is necessary in clinical and environmental toxicology. Leaf extracts of *Lycoris longituba* were used as reducing agent for the one-step synthesis of reduced graphene oxide-Ag nanocomposites. This nanocomposite was characterized by TEM, FTIR, XRD, and other instruments. Then, the graphene/Ag nanocomposite was used as a modifier to be cemented on the surface of the glassy carbon electrode. This electrode exhibited excellent electrochemical sensing performance. Under the optimal conditions, the proposed electrode could detect imatinib at 10 nM−0.28 mM with a low limit of detection. This electrochemical sensor also has excellent anti-interference performance and reproducibility.

## Introduction

Cancer is one of the most important diseases facing humanity today. Cancer is lethal because its cells are uncontrolled and proliferate indefinitely and spread throughout the body. Cytostatic agents are the most commonly used class of anti-cancer drugs (Karthik et al., 2017; Liu et al., 2018; Muti and Muti, 2018; Zahed et al., 2018). Their purpose is to inhibit the growth of cancer cells. However, the widespread use of cytostatic agents has caused some other effects, such as on environmental toxicology. Among them, imatinib is a specific inhibitor. Imatinib is frequently used in the treatment of chronic myeloid leukemia and gastrointestinal stromal tumors (Cahill et al., 2017; Hochhaus et al., 2017). For example, patients with chronic myeloid leukemia can use 400 mg of imatinib per day. Previous reports have demonstrated that the cytogenetic and molecular response to imatinib is associated with low plasma concentrations in patients with chronic myeloid leukemia (Serrano et al., 2019; Buclin et al., 2020). Therefore, monitoring of imatinib is necessary, both in the clinical and environmental toxicology fields.

Currently, the detection methods for imatinib include UV-vis spectroscopy (Grante et al., 2014), HPLC (Roth et al., 2010), and electrophoresis (Li et al., 2012; Ahmed et al., 2019). Although these methods can be used for the rapid detection of imatinib, they all have their own drawbacks. For example, UV-vis spectroscopy requires a large number of samples. HPLC methods are slower and require large instruments. The detection sensitivity of electrophoresis method is not enough. Therefore, it is necessary to develop a technique for the rapid detection of imatinib. Electrochemical sensors are a fast and sensitive detection technology. It enables highly sensitive detection of electrochemically active substances. Previous studies have demonstrated that imatinib can be oxidized at lower potentials, so assembling an imatinib-based electrochemical sensor is an approach worth investigating.

Conventional electrochemical sensors use carbon electrodes for the detection of target molecules (Fu et al., 2019; Mahmoudi-Moghaddam et al., 2019; Zhou et al., 2020). Carbon electrodes have a stable electrochemical window and do not react easily with other substances. However, the electrochemical signal of ordinary carbon electrodes is weak (Karimi-Maleh et al., 2021a), so it is difficult to meet the demand of highly sensitive detection. Modification on the surface of ordinary carbon electrodes is a common method to improve the electrochemical activity of sensors (Cao et al., 2019; Alam et al., 2020; Fu et al., 2020). Recent studies have shown that modification of carbon nanomaterials on the surface of glassy carbon electrodes can improve the performance (Karimi-Maleh et al., 2020). For example, modification of graphene on the surface of glassy carbon electrodes can increase the electrical conductivity. However, the layer-layer interaction of graphene is so strong that direct modification can cause agglomeration, which in turn reduces the performance of the sensor (Kumar et al., 2019; Jadoon et al., 2020). Surface modification of polymers allows the surface of graphene to be loaded with tubular energy groups. Under the principle of homogeneous charge repulsion, the layer-layers of graphene can be separated from each other. However, the modified graphene also cannot perform very well due to the poor electrical conductivity of the polymer (Liu et al., 2019). Another strategy is to grow nanoparticles between graphene. The graphene lamellae are separated by nanoparticles. This approach is most beneficial for the modification of electrochemical sensors (Karimi-Maleh et al., 2021b). The presence of nanoparticles can increase the electrochemically active surface area. Also, some nanoparticles have electrochemical catalytic properties that can improve the sensitivity of detection.

Graphene-nanoparticle composites are synthesized by many methods. In recent years, the one-step synthesis of graphene-nanoparticle composites using plant extracts has attracted much attention (Nandgaonkar et al., 2014; Keerthi et al., 2018; Khanam and Hasan, 2019). This method does not require polluting reducing agents and easily controls the size of nanoparticles. For example, Song and Shi (2019) reported the synthesis of graphene/Ag nanocomposites using *Shewanella oneidensis*. Weng et al. (2018) reported the synthesis of graphene/Fe nanocomposites using eucalyptus leaves.

In this work, we chose the leaf extract of *Lycoris longituba* as a reducing agent. The graphene/Ag nanocomposites were reduced by a one-step hydrothermal method. We characterized the conforming materials. The synthesized composites were used for surface modification of glassy carbon electrodes and successfully used for electrochemical detection of imatinib. This novel electrochemical sensor allows highly sensitive detection of imatinib.

## Experiments

All reagents, including KH_2_PO_4_, Na_2_HPO_4_ and silver nitrate were purchased from Macklin Co. Ltd. and used without purification. Graphene oxide (GO) was purchased from Nanin Youshan Biotech Co. Ltd. *Lycoris longituba* was purchased from local nursery. The working electrode, counter electrode and reference electrode were glassy carbon electrode (GCE), Pt wire and Ag/AgCl (3M), respectively. Phosphate buffer solution (PBS) was prepared by mixing stock solutions of 0.1 M disodium hydrogen phosphate and sodium dihydrogen phosphate. The electrochemical determination of imatinib was carried out using a CHI760 electrochemical workstation. Differential pulse voltammetry (DPV) was used for electrochemical recording. The scan range was 0–1.2 V. The pulse amplitude was 50 mV. The pulse width was 0.05 s. The pulse period was 0.5 s.

The XRD pattern of sample was collected by a XRD with Cu Kα (λ = 0.1546 nm) radiation (D8-Advanced, Bruker). Transmission electron microscopy (TEM) image was observed with a JEOL JEM-2100 high-resolution transmission electron microscope. FTIR spectra were collected by a Fourier transform infrared spectroscopy (Nicolet iS5, Thermo Scientific).

To prepare the aqueous extract of *Lycoris longituba*, its crushed leaf (1:20 ratio) was shaken overnight at 200 rpm with water as solvent. Then, the mixture was filtered, and the resulting extract was used for further experiment. Then, 5 mL of the extract was diluted to 20 mL, and silver nitrate (10 mM) was added to it. This solution was transferred to an autoclave and heated at 120°C for 10 h. The composite was collected after filtration and re-dispersed into water to form a 0.5 mg/mL dispersion (denoted as G/Ag).

## Results and Discussion

**Figure 1:**
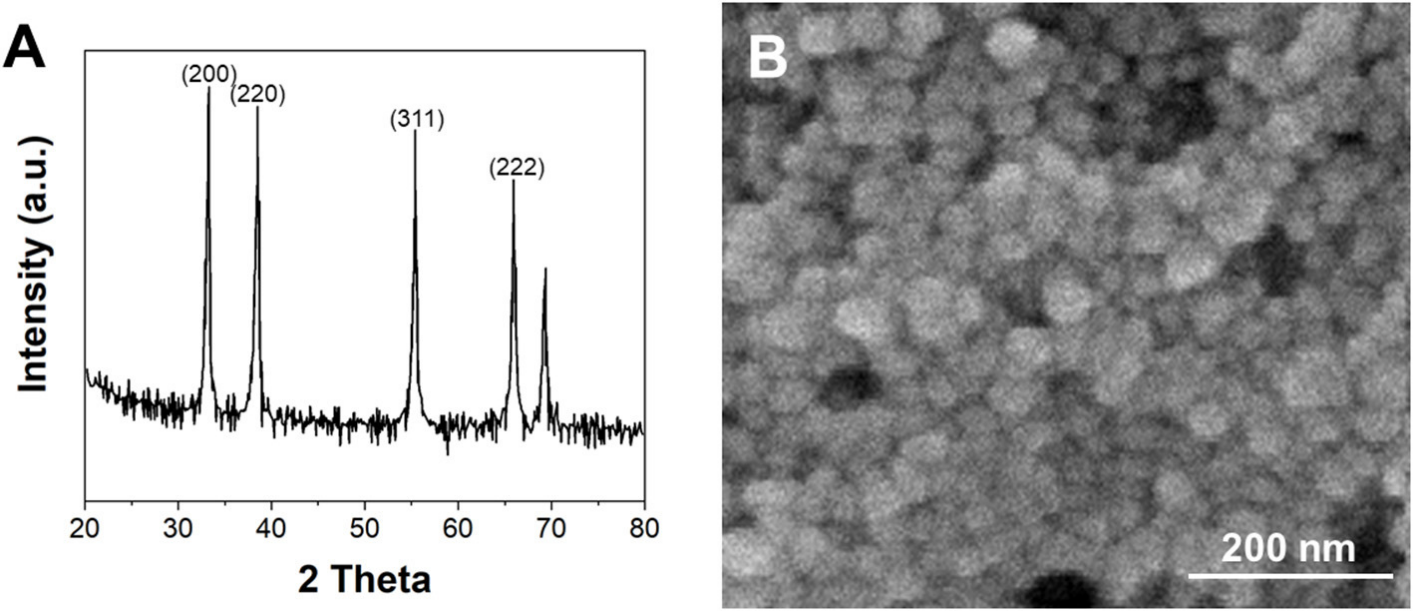
**(A)** XRD pattern and **(B)** TEM image of biosynthesized G/Ag.

Figure 1A shows the XRD pattern of the synthesized G/Ag. It can be seen that four distinct planes of the sample corresponding to (200), (220), (311), and (222) lattice plane of silver face-centered-cube (fcc) crystal (Waterhouse et al., 2001). This result was mated to the reference data in JCPDS file no. 04-0783, suggesting the successful formation of the Ag nanoparticles. Moreover, an additional peak appearing at 26.10° can be also noticed, due to the partial reduced GO sheets to form an ordered crystalline structure (Wang et al., 2013). Figure 1B shows the TEM image of the synthesized G/Ag. By the contrast with the background, we can see the layered graphene. On the graphene lamellae we can see the growing Ag nanoparticles. According to the statistics, the average size of Ag nanoparticles is 24 nm.

**Figure 2:**
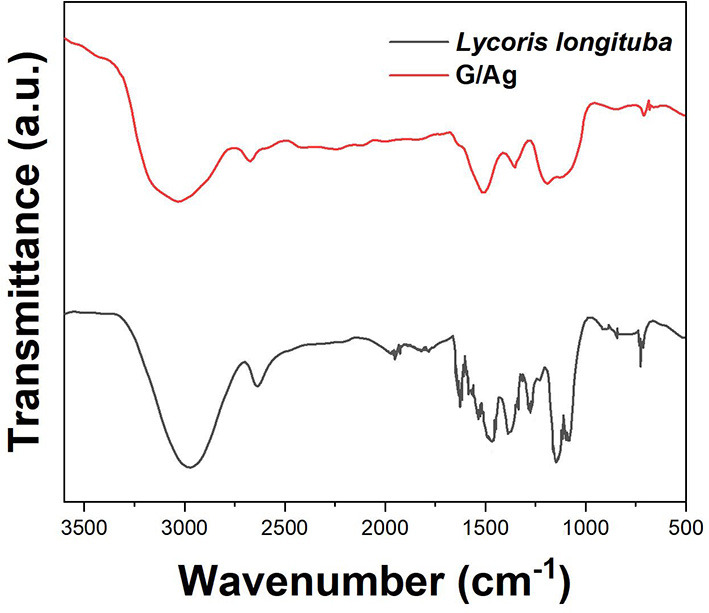
FTIR spectrum of *Lycoris longituba* leaf extract and G/Ag.

Figure 2 shows the FTIR spectra of *Lycoris longituba*, and biosynthesized G/Ag. It can be seen that the extract of *Lycoris longituba* exhibited a series bands at range from 700 to 2,000 cm^−1^. The absorbance peak at 1,322 cm^−1^ is corresponding to the C–O stretching (Ranjana and Mendhulkar, 2015). In addition, the peak located at 883 cm^−1^ can be assigned to the C–N vibrations of the nitroso groups. These two peaks were also found in the biosynthesized G/Ag, suggesting the biomolecules of the *Lycoris longituba* were attached on the composite surface. The intensity of the oxygen containing groups on the G/Ag is relatively low, indicating the reduction of GO during the hydrothermal treatment.

**Figure 3:**
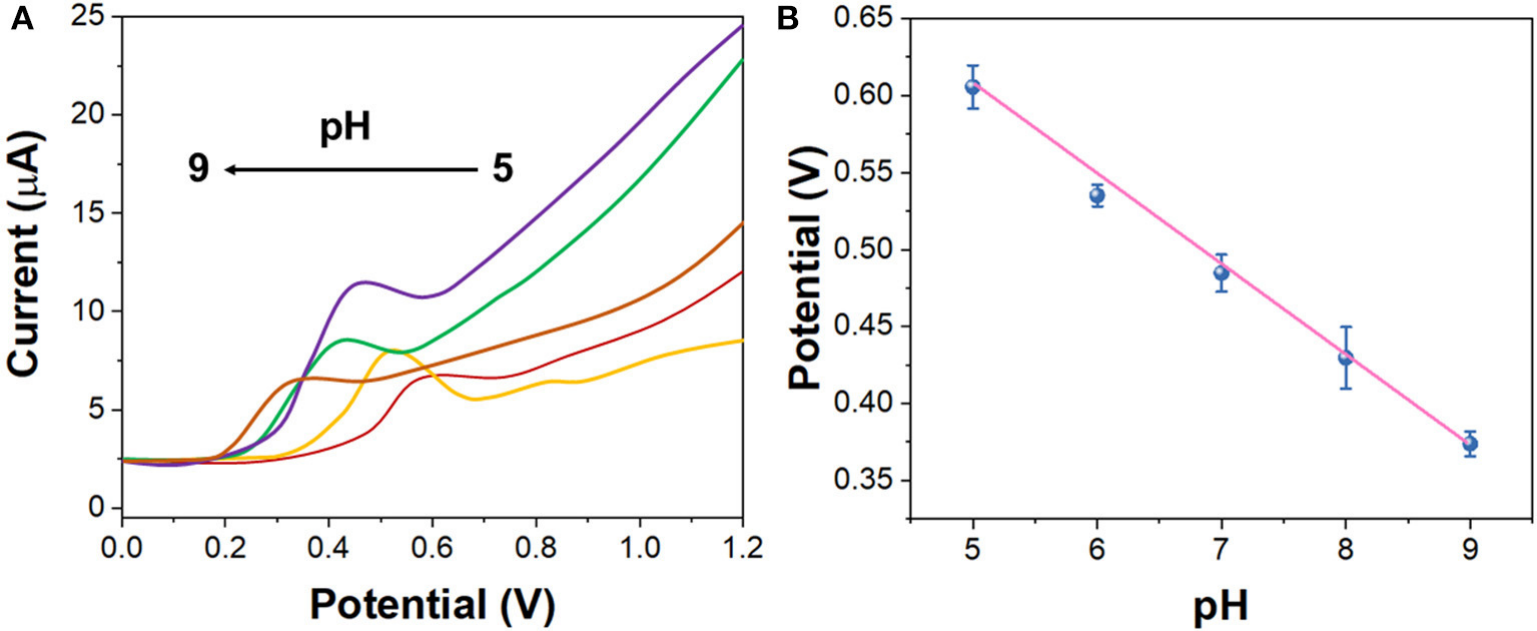
**(A)** DPV curves of the G/Ag/GCE in 10 μM imatinib solution in the pH range between 4.0 and 8.0. **(B)** Plots of oxidation potential of imatinib vs. pH value.

Figure 3A shows the DPV curves of G/Ag/GCE in 10 μM imatinib solution in the pH range between 4.0 and 8.0. It can be seen that the oxidation potential of imatinib shifted to positive direction along with the increase of the pH. Figure 3B shows the plot of oxidation potential of imatinib vs. pH value. It can be seen that a slope of 56.9 mV/pH was obtained, suggesting the equal number of electron and proton were participated in the reaction. In addition, the maximum oxidation response for imatinib was observed at pH = 7.0. Therefore, pH = 7.0 was selected as the optimal condition.

**Figure 4:**
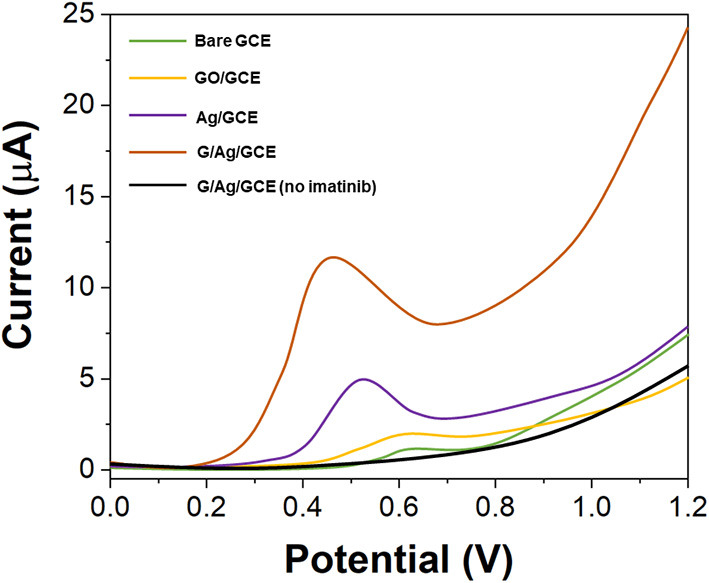
DPV curves of the bare GCE, GO/GCE, Ag/GCE, and G/Ag/GCE toward 10 μM imatinib.

Figure 4 shows the DPV curves of 10 μM imatinib using bare GCE, GO/GCE, Ag/GCE, and G/Ag/GCE. It can be seen that the GCE only exhibited a very small oxidation peak with a current response of 1.34 μA. The modification of GO on the GCE showed no clear enhancement. In contrast, the modification of Ag nanopartciles on the GCE surface showed an excellent performance toward imatinib oxidation. An enhanced oxidation peak was observed with 4.52 μA response, suggesting the good electrical conductivity of Ag nanoparticles can enhance the sensing performance. In addition, the G/Ag/GCE showed an even higher response toward imatinib with a much lower oxidation potential, suggesting the combination of Ag and reduced GO can trigger the electrocatalytic reaction with imatinib. The electrocatalytic activity of the Ag nanoaprtciles was observed when the corporation with carbon based materials (Asadian et al., 2017; Liu et al., 2017; Majidi and Ghaderi, 2017; Kumar and Goyal, 2018).

**Figure 5:**
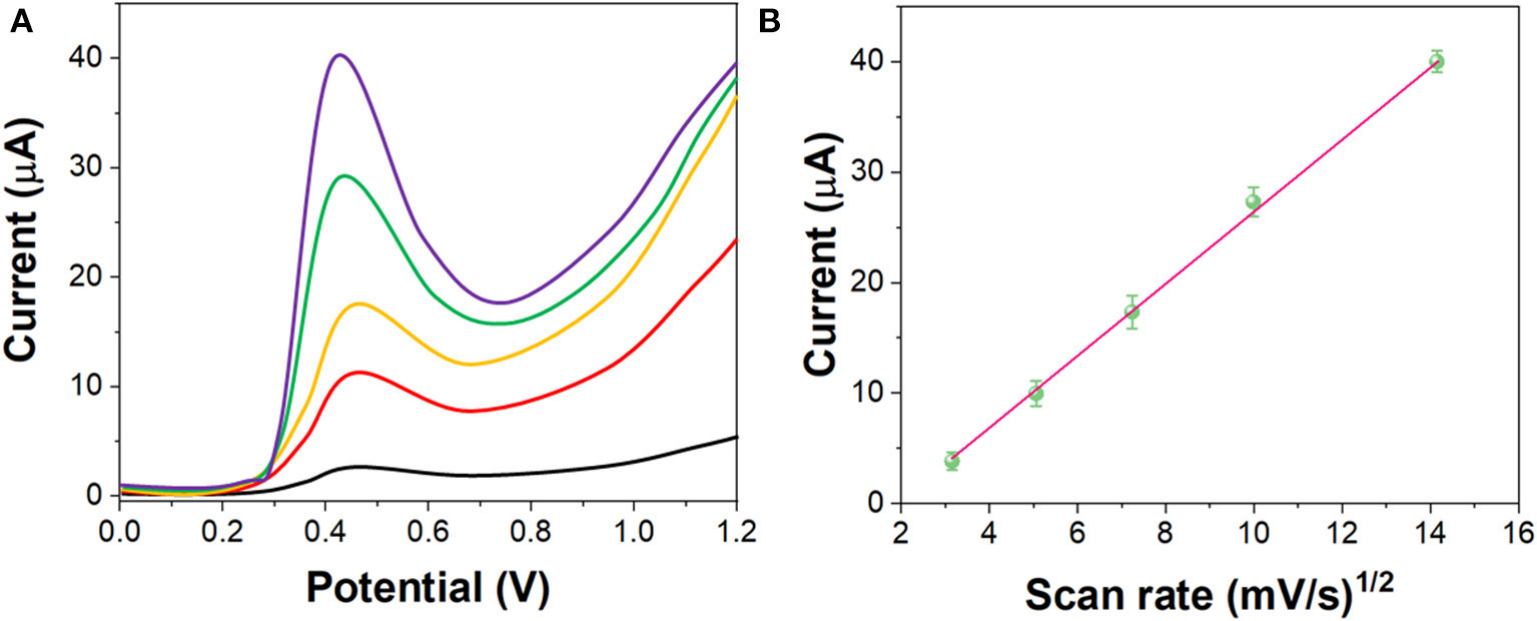
**(A)** LSV curves of the G/Ag/GCE in 10 μM imatinib solution in the scan rate between 10 and 200 mV/s. **(B)** Plots of v^1/2^ vs. current response.

Figure 5 shows the LSV curves of G/Ag/GCE toward 10 μM imatinib with different scan rate (from 10 to 200 mV/s). It can be seen that, the electrochemical oxidation current of the imatinib had a liner relationship with the v^1/2^ while the oxidation potential shifted positively along with the increase of the scan rate, indicating the electrochemical behavior of the imatinib obeyed the diffusion-controlled process (Ivanishchev et al., 2016).

**Figure 6:**
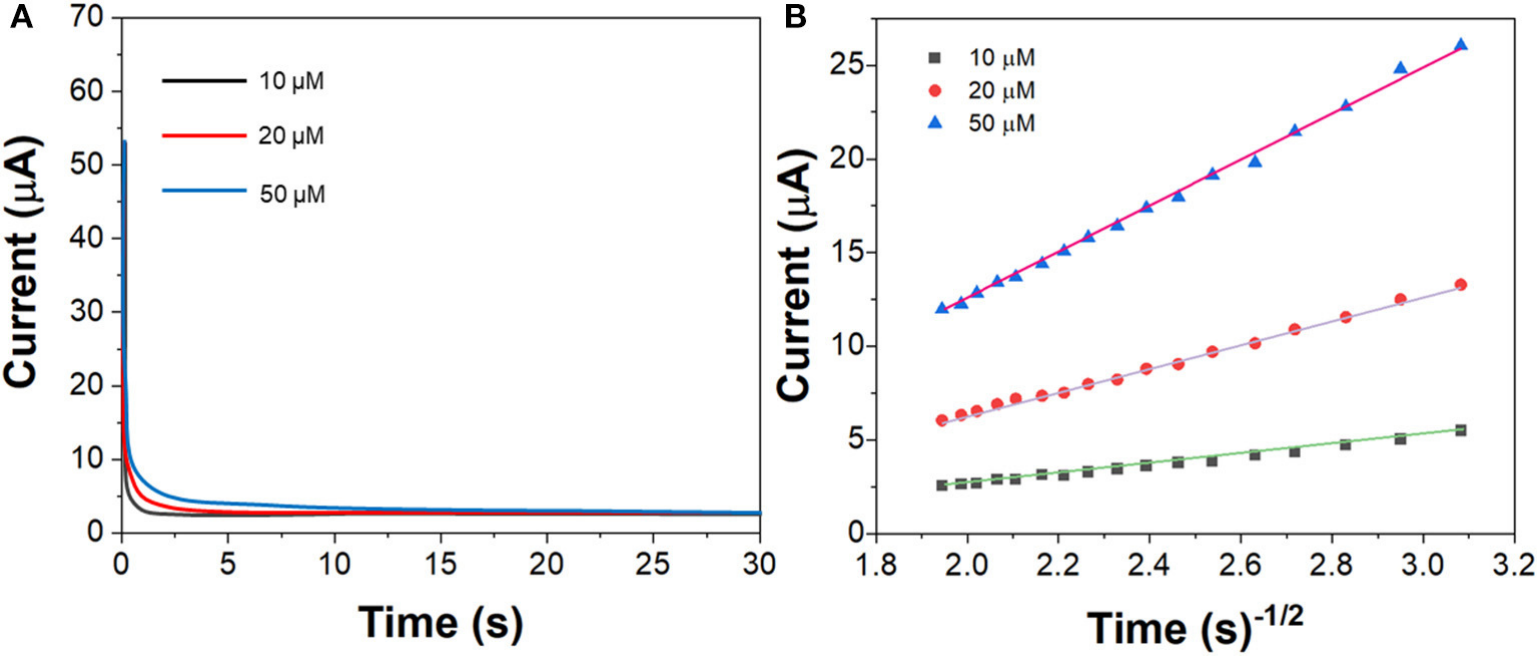
**(A)** Chronoamperograms of the G/Ag/GCE in imatinib solution in the concentration of 10, 20, and 50 μM. **(B)** Plots of t^−1/2^ vs. current response.

Figure 6A shows the chronoamperograms of G/Ag/GCE toward imatinib with 10, 20, and 50 μM. Figure 6B shows the corresponded cottrell plots. The diffusion coefficient was calculated to be ~1.94 × 10^−5^ cm^2^/s.

**Figure 7:**
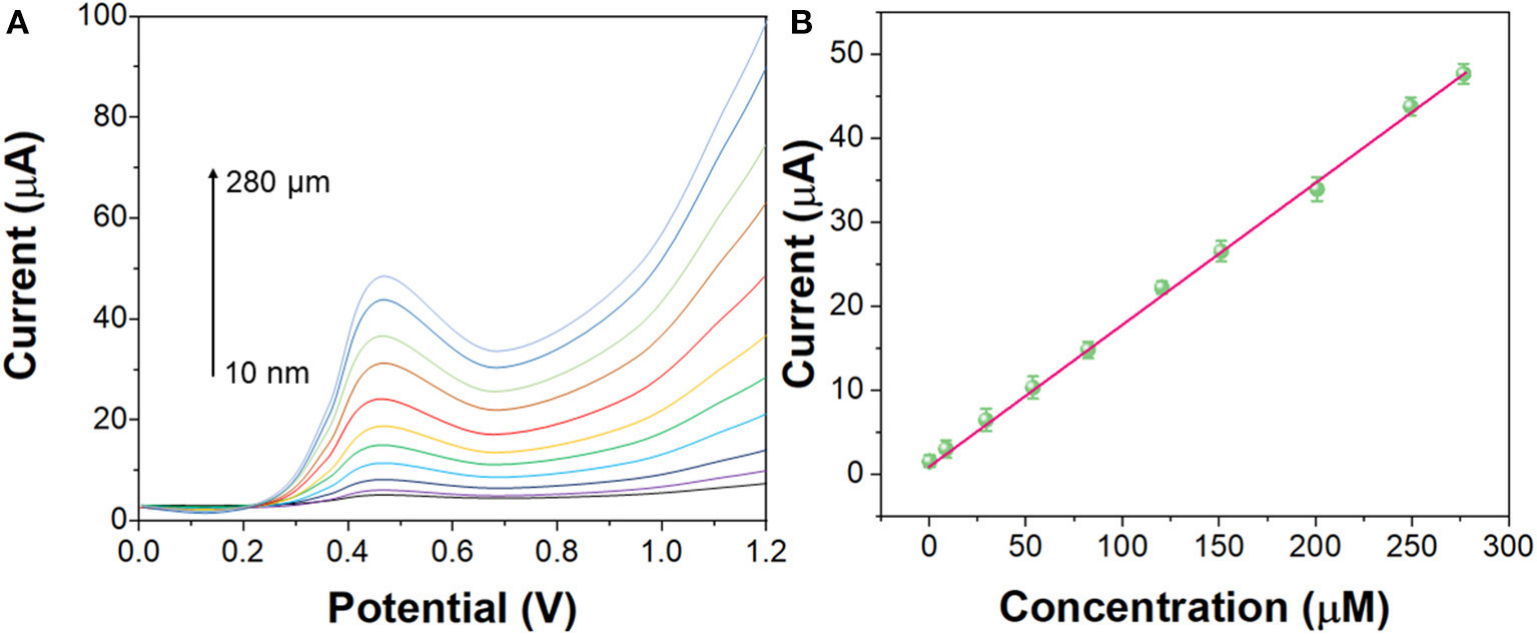
**(A)** DPV curves of the G/Ag/GCE in imatinib solution in the concentration from 10 nM to 280 μM. **(B)** Linear calibration plot of G/Ag/GCE toward concentration of imatinib.

As shown in Figure 7, under the optimal experimental conditions, the DPV curves of G/Ag/GCE for different concentrations of imatinib were recorded. G/Ag/GCE has a linear relationship with the concentration of imatinib, with a linear range of 10 nM−280 μM and a detection limit of 1.1 nM (S/N = 3). As shown in Table 1, compared with other methods, this method has a lower detection limit and a wider linear range. Although there are some detection methods that work better than our proposed sensors, such as LC/MS/MS and LC/TMS, these methods require large instruments and cannot achieve rapid detection.

**Table 1:** Electrochemical imatinib sensor performance comparison.

**Method**	**Linear detection range**	**Detection limit**	**Reference**
SWV	19 nM−1.9 μM	5.55 nM	Chen et al., 2014
MS	0.1 nM−1 μM	–	Friedecký et al., 2015
DPV	10 nM−200 μM	7.39 nM	Hatamluyi and Es'haghi, 2017
DPV	30 nM−0.25 μM	6.3 nM	Brycht et al., 2016
LC/MS/MS	100 nM−7.091 μM	0.1 μM	Andriamanana et al., 2013
Liquid chromatography-tandem mass spectrometry	20 nM−2.052 μM	–	Yang et al., 2013
DPV	10 nM-280 μM	1.1 nM	This work

**Figure 8:**
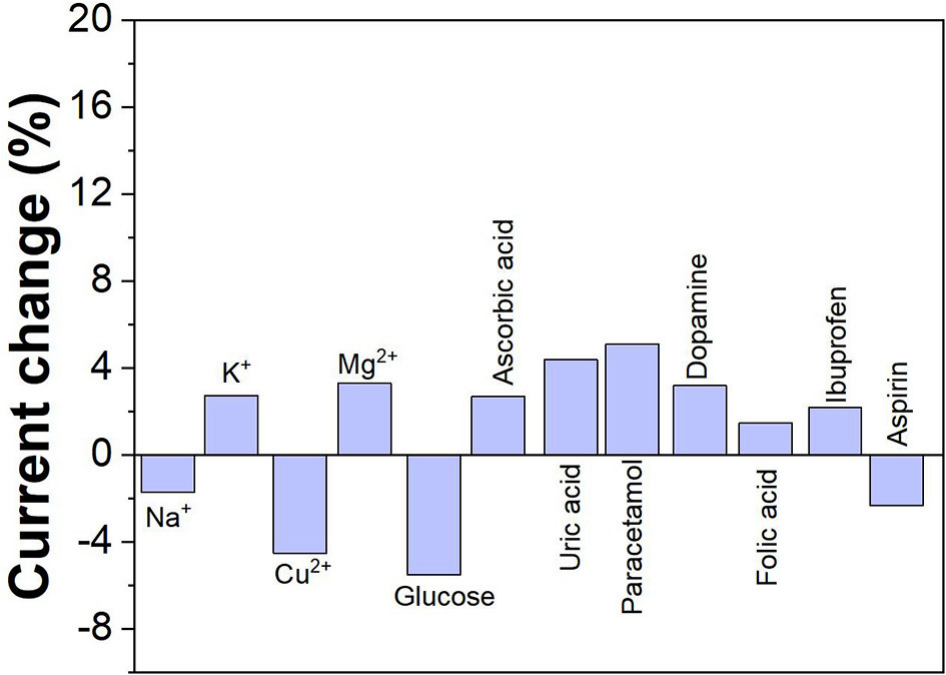
Anti-interference property of the G/Ag/GCE.

In order to discuss the selectivity of G/Ag/GCE, 13 common ions that may interfere with the actual detection of imatinib were investigated in this experiment, as shown in Figure 8. The results show that when the concentration of sodium ion, potassium ion, copper ion, manganese ion, cobalt ion, magnesium ion, mercury ion, zinc ion, lead ion, nickel ion, barium ion, aluminum ion and chromium ion is 10 times higher than that of imatinib, there is no obvious interference to the actual detection of imatinib. Therefore, the proposed sensor exhibited an excellent anti-interference property.

The proposed sensor has been then used for determining the content of imatinib in serum samples. Standard addition of imatinib was used. Table 2 shows the performance of the sensor for real sample analysis. It can be seen that excellent recovery performance was observed for each test, indicating the proposed sensor can be applied for real sample sensing.

**Table 2:** Electrochemical determination of imatinib content in serum samples using G/Ag/GCE.

**Sample**	**Detected (nM)**	**Added (nM)**	**Detected (nM)**	**Recovery (%)**
1	0	10.00	9.77	97.70
2	0	20.00	20.47	102.35
3	0	50.00	50.44	100.88
4	0	100.00	99.36	99.36

## Conclusions

The stable Ag nanoparticles were synthesized with the reduction of GO using leaf extracts of *Lycoris longituba* under hydrothermal condition. TEM, XRD and FTIR were used for characterizations. Based on the enhancement effect of imatinib on the electrochemical oxidation signal, an electrochemical sensor was constructed using G/Ag nanocomposite. Under the optimal experimental conditions, a linear range of imatinib detection was obtained between 10 nM and 280 μM with a limit of detection of 1.1 nM. The proposed G/Ag/GCE showed an excellent anti-interference property. In addition, it can be applied to the detection of imatinib in real serum sample.

## Data Availability Statement

The original contributions presented in the study are included in the article/supplementary material, further inquiries can be directed to the corresponding author/s.

## Author Contributions

ZW, YW, and JL conceived of the study. YW and ZW supervised the development program, collected materials characterization. ML, CZ, and HZ received and curated samples and analytical records. HZ, ZW, and YW wrote the manuscript. All authors read and approved of the manuscript.

## Conflict of Interest

The authors declare that the research was conducted in the absence of any commercial or financial relationships that could be construed as a potential conflict of interest.
